# Insulin Resistance Is Associated with Multiple Chemical Sensitivity in a Danish Population-Based Study—DanFunD

**DOI:** 10.3390/ijerph182312654

**Published:** 2021-11-30

**Authors:** Anne A. Bjerregaard, Marie W. Petersen, Lise Kirstine Gormsen, Sine Skovbjerg, Niklas R. Jørgensen, Allan Linneberg, José G. Cedeño-Laurent, Torben Jørgensen, Thomas M. Dantoft

**Affiliations:** 1 Center for Clinical Research and Prevention, Bispebjerg and Frederiksberg Hospital, Nordre Fasanvej 57, Hovedvejen, Entrance 5, 2000 Frederiksberg, Denmark; allan.linneberg@regionh.dk (A.L.); Torben.joergensen@regionh.dk (T.J.); Thomas.meinertz.dantoft@regionh.dk (T.M.D.); 2 Department of Epidemiology Research, Statens Serum Institut, Artillerivej 3, 2300 Copenhagen, Denmark; 3 Research Clinic for Functional Disorders and Psychosomatics, Aarhus University Hospital, Universitetsbyen 22–23, 8000 Aarhus C, Denmark; mawept@rm.dk (M.W.P.); lisgor@rm.dk (L.K.G.); 4 The Danish Center for Mindfulness, Department of Clinical Medicine, Aarhus University, Hack Kampmanns Plads, 8000 Aarhus C, Denmark; sine.skovbjerg@clin.au.dk; 5 Department of Clinical Biochemistry, Rigshospitalet, Valdemar Hansens Vej 13, 2600 Glostrup, Denmark; niklas.rye.joergensen@regionh.dk; 6 Institute of Clinical Medicine, University of Copenhagen, Blegdamsvej 3, 2200 Copenhagen, Denmark; 7 Department of Clinical Medicine, Faculty of Health and Medical Sciences, University of Copenhagen, Blegdamsvej 3B, 2200 Copenhagen, Denmark; 8 Department of Exposure Epidemiology and Risk Program, Harvard T.H. Chan School of Public Health, 1350 Massachusetts Ave, Cambridge, MA 02138, USA; jcedenol@hsph.harvard.edu; 9 Department of Public Health, Faculty of Health and Medical Sciences, University of Copenhagen, Blegdamsvej 3B, 2200 Copenhagen, Denmark

**Keywords:** multiple chemical sensitivity, MSC, DanFunD, functional somatic disorders

## Abstract

Multiple chemical sensitivity (MCS) is a multisystem syndrome, and limited knowledge of its pathophysiology exists. Based on the population-based Danish cohort *DanFunD*, this study investigated metabolic health in people with MCS compared to individuals who did not have MCS. From 9656 cohort participants aged 18–76 years old, 1.95% were categorized as MCS individuals with comorbid functional somatic disorders (MCS *+* *FSD*, *n* = 188), and 1.13% were categorized as MCS without functional somatic disorders (MCS *÷* *FSD*, *n* = 109). MCS was characterized based on three criteria: the experience of symptoms upon exposure to common odors and airborne chemicals, symptoms related the central nervous systems and others organ symptoms, and significant impact on every day, social, and occupational life. The remaining study population without MCS or any other functional somatic disorders were regarded as controls. We used adjusted multiple linear regression with link-function to evaluate the associations between lipid and glucose metabolism markers and MCS. We also tested the odds ratio of metabolic syndrome in MCS. Results did not point to statistically significant associations between lipid biomarkers or metabolic syndrome and both MCS groups compared to the controls. We found that MCS individuals may be more insulin resistant and that MCS ÷ *FSD* may have an impaired glucose metabolism when compared to controls.

## 1. Introduction

Multiple chemical sensitivity (MCS) is a multisystem syndrome that affects between 0.5–6.5% of the population [1,2,3,4,5]. By some, MCS is characterized as a functional somatic disorder (*FSD*), with individuals experiencing a wide range of symptoms that are attributed to airborne chemical exposures that most people consider benign. MCS patients often experience symptoms that are related to the central nervous system (CNS), e.g., headache, fatigue, nausea, or other physical reactions often involving several organ systems, e.g., the gastro-intestinal, the respiratory, or the cardio-vascular system [2,6]. Thus, the disorder has a substantial impact on the everyday, social, and occupational lives of those who are affected [7,8]. The symptoms often overlap with the symptoms of *FSD*s, including irritable bowel syndrome (IBS), fibromyalgia, and chronic fatigue syndrome, and MCS individuals often fulfill the diagnostic criteria for several *FSD*s [9]. Collectively, this contributes to the complex matrix of symptoms and comorbidities within MCS and increases the challenge of studying the epidemiology.

In population-based studies, the majority of MCS individuals is women, many are often unemployed, and of lower social status [2,6,10,11,12]. Moreover, MCS individuals were found to have a more sedentary lifestyle, experience daily physical limitations, and sleep disturbances [2]. In general, MCS constitutes a highly heterogenic group in relation to exposure triggers, symptoms, and illness severity where extremely low exposure levels to common airborne chemicals may elicit symptoms. However, traditional toxicological dose–response relationship between exposure levels and the elicitation of symptoms or symptom severity does not exit [13]. 

Several theories on the pathophysiology of MCS have been investigated, including increased responses in the CNS, the impairment of the immune, olfactory, and respiratory systems, behavioral conditioning, and psychiatric disorders [14,15]. The organ systems affected in MCS and *FSD* are the same organs that perform important processes to maintain optimal metabolic health. Nevertheless, disturbances in metabolic health and the examination of more traditional biochemical markers in relation to the pathophysiology of MCS is poorly described in the literature. One study found differences in the fatty acid profile in the erythrocyte membranes when comparing MCS patients with controls [14], whereas another study found no differences in fasting glucose, insulin, or insulin resistance (measureed by HOMA-index) [16]. When looking into other *FSD*s, metabolic disturbances have been reported in both fibromyalgia, chronic fatigue syndrome, and IBS, which were found to be associated with metabolic syndrome when comparing cases with healthy controls [17,18,19]. However, no studies have reported whether metabolic syndrome or other metabolic disturbances other than those mentioned above are associated with MCS beyond what could be related to comorbid *FSD*s. Such knowledge can provide useful information to the pathophysiology of MCS.

The aim of this study was to investigate metabolic health in Danish MCS individuals and more specifically, to examine the lipid and glucose metabolism compared to individuals without MCS. 

## 2. Materials and Methods

### 2.1. Study Population

The analyses are based on data from The Danish Study of Functional Disorders (DanFunD). Study details have been described elsewhere [20]. In short, the DanFunD study is a cohort study comprised a random sample of 9656 men and women aged 18–76 years and living in 10 municipalities in the western part of greater Copenhagen at baseline. All participants completed a general health examination and completed an extensive questionnaire battery. 

Based on standardized validated questionnaires, five different *FSD*s were delimited [21], and the participants fulfilling criteria for MCS [22,23], chronic widespread pain [24], chronic fatigue [25,26], irritable bowel [27], and whiplash-associated disorders [28] were identified [20]. The case criteria for MCS were constructed as a reduced adaptation of the 1999 US Consensus Criteria for MCS and the revisions suggested by Lacour and colleagues [22,23]. Lacour and colleagues suggested that MCS cases are defined based on six criteria: (1) it is a chronic condition (of at least 6 months), (2) reproducible symptoms recur, (3) multiple organ systems are involved (CNS is obligatory along with at least one other system), (4) symptoms occur in response to low levels of exposure, (5) multiple unrelated chemicals trigger symptoms, and (6) symptoms improve or resolve when exposure to the trigger agents is removed [22]. Thus, in the present study, the following three criteria needed to be fulfilled:

1. The participant needed to have experienced symptoms upon being exposed to at least 2 of 11 common odors and airborne chemicals; and
2. The parcipant needed to have experienced at least one symptom from the central nervous system and at least one symptom from another organ system in response to the inhalation of airborne odors or chemicals; and
3. The participant needed to report significant lifestyle or functional impairment due to the symptoms related to the inhalation of airborne chemicals and odor, which could be defined as responding affirmably to the statements (A) symptoms have influenced my choice of products used for personal hygiene, products used for cleaning at home, and/or the choice of places where I do my daily shopping and either (B) symptoms have negatively influenced my social lifestyle (i.e., limited my possibility to use public transport, dine at restaurants, go to the cinema/theatre, participate in meetings/social events) or (C) symptoms have negatively influenced my occupational condition (i.e., have had to go on temporary sick leave, discontinued education/employment, been unable to hold a job position, or unable to complete my education).

From the full DanFunD study population, we identified all of the participants fulfilling the criteria for MCS. From these participants, two subgroups were created, i.e., individuals fulfilling the MCS criteria and none of the additional four *FSD*s screened for (MCS ÷ *FSD*) and MCS individuals fulfilling the criteria for MCS and at least one of the additional four *FSD*s screened for in the DanFunD study (MCS + *FSD*). The remaining study population, participants without any *FSD* comorbidities, were categorized as the control group with no *FSD* in analyses.

### 2.2. Assessment of Biomarkers

Fasting blood plasma samples were drawn during the health examination, which were analyzed for lipids (total cholesterol, HDL-cholesterol, and triglycerides) and glucose, insulin, and glycated haemoglobin (HbA1c). Lipids were measured using colorimetric slide methods with Vitros 4600/5600 Ortho Clinical Diagnostics (OCD), and non-HDL cholesterol was calculated by using the equation: Total cholesterol − HDL-cholesterol. Glucose was assessed by the hexokinase/glucose-6-phosphate dehydrogenase assay (Roche Diagnostics, former Boehringer Mannheim, Germany), insulin was assessed by the flouroimmunoassay technique, AutoDelfia (Perkin Elmer-Wallac, Finland), and HbA1c was assessed by the HPLC method (TOSOH, Minato, Japan). The homeostasis model of insulin resistance, HOMA-IR, was calculated using the formula: ((fasting plasma insulin [pmol/L]* fasting plasma glucose [pmol/L]/22.5) × 0.144) [29]. Based on the concept that blood glucose and insulin concentrations are related by the feedback of glucose on beta-cells to increase insulin secretion, this model is used as a surrogate measure of insulin sensitivity [29].

### 2.3. Lifestyle Factors and Physiological Assessments

Information on general health, mental vulnerability, and other lifestyle factors were collected via validated questionnaires. Self-perceived health was measured with a single question from the 12-item Short Form Health Survey (SF-12) [30] on a five-point Likert scale from “excellent” to “poor”. Subsequently, self-perceived health was dichotomized into “poor self-perceived health” (fair/poor) and “good self-perceived health” (excellent/very good/good). A similar method and procedure were used for self-perceived fitness, which was dichotomized into “very good self-perceived fitness” and “poor self-perceived fitness” [31]. Self-perceived stress was assessed using Cohen’s Perceived Stress scale [32], and mental health including anxiety and depression was assessed using the Symptom Checklist (SCL) ANX-10 and DEP-13, respectively [33]. Additionally, information on heart diseases and treatment were also retrieved from questionnaires asking “Have you ever been told by a physician that you have or have had myocardial infarction or other heart diseases?” and “Do you receive treatment for diabetes, high blood pressure or high cholesterol?”. 

All physiological measurements were conducted by trained nurses using standardized protocols. Height (cm) was measured once without shoes, and weight (kg) and body fat percentage were measured once while the participant was wearing light clothes (fasting) using a Tanita TBF-300 body composition analyzer. Subsequently, BMI was calculated as weight (kg)/height (m)^2^, and a BMI between 18 to ≤25 kg/m^2^, 25 to ≤29 kg/m^2^, and ≥30 kg/m^2^ was categorized normal weight, overweight, and obese, respectively. Waist circumference (cm) was measured once by placing a measuring tape midway between the lower rib curvature and the upper hip edge. 

Metabolic syndrome was defined in accordance with the criteria of the American Heart Association scientific statements of 2021 [34]. Participants were considered to have metabolic syndrome when they presented three or more of the following components: (1) elevated waist circumference of >102 cm in men and >88 cm in women, (2) elevated triglycerides (≥1.7 mmol/L) or drug treatment for elevated triglycerides, (3) reduced HDL-cholesterol (<1.04 mmol/L in men; women <1.30 mmol/L) or drug treatment for reduced HDL-cholesterol, (4) elevated blood pressure (SBP ≥ 130 mm Hg and/or DBP ≥ 85 mm Hg) or antihypertensive drug treatment, (5) elevated fasting glucose (≥6.1 mmol/L) or drug treatment of elevated glucose.

### 2.4. Statistical Analyses

Standard descriptive statistics were applied to describe distributions (mean (±SD)/median (5th and 95th percentiles)/*n*(%)) within MCS + *FSD*, MCS ÷ *FSD*, and the control group. Mean/median differences for continuous variables with normal or skewed distributions were compared between MCS + *FSD*, MCS ÷ *FSD* and the control group, respectively using a T-test (ANOVA) or the Mann–Whitney test. The chi-squared (*X*^2^) test was applied for categorical variables.

Multiple linear regression models for continuous outcomes used a link function, and the results were presented as the percent difference in each outcome. To test whether the assumptions for linear regression analyses were met, residuals for normal distribution and homogeneity of variance were visually examined. A priori, we tested the interaction between age and sex and between MCS and age for all of the dependent variables and included age and sex adjustment in the crude model (model 1). In model 2, we further adjusted for physical activity (METS, continuous), BMI (continuous), and waist circumference (continuous). In model 3, we further adjusted for Cohens perceived stress scale (continues). To test the effect modification of chronic stress, the interaction between outcomes and Cohens perceived stress scale was examined in model 1. The measures for the lipid markers that were taken in models 1 and 3 subsequently excluded participants with known diabetes and statin users. Participants with known diabetes were excluded a priori from analyses determining glucose, HbA1c, insulin, and HOMA-IR, and subsequently, models 1 through 3 were performed while excluding statin users as well. In the sensitivity analyses, we further adjusted model 2 for any known heart diseases, SCL-depression, and anxiety as well as self-perceived health status and self-perceived physical fitness.

A logistic regression was applied to model 1, which excluded individuals with diabetes, and model 2, which also excluded statin users, to test the odds of metabolic syndrome in MCS compared to the control group. Both models were first adjusted for age and sex, and then they were further adjusted for physical activity and BMI. The best model fit was tested as it was by Hosmer and Lemeshow, which was achieved by including age to the power of two. 

Statistical analyses were performed using SAS Enterprise guide 7.15. Results are assessed at a 5% significance level.

## 3. Results

The prevalence (95%CI) of MCS + *FSD* and MCS ÷ *FSD* in the DanFunD cohort (*n* = 9656) was 1.95% (1.67–2.22) and 1.13% (0.92–1.33), respectively. Characteristics of the participants categorized into MCS + *FSD* (*n* = 188), MCS ÷ *FSD* (*n* = 109) and the control group (*n* = 7791) and the levels of biochemical markers are displayed in Table 1. Among MCS + *FSD* and MCS ÷ *FSD*, a higher proportion of the participants was women compared to the controls (*p* < 0.001 and *p* = 0.03), and the participants in the MCS ÷ *FSD* group were slightly older than the controls on average (*p* = 0.04). Compared to the control group, MCS + *FSD* had significantly elevated BMI and fat percentage, and more were categorized as obese. Further, the MCS + *FSD* group had a higher level of stress, anxiety, and depression and lower self-perceived health and fitness (*p* < 0.0001). Comparing MCS ÷ *FSD* with the control group showed similar differences regarding fat percentage, obesity, stress, anxiety, depression, and self-perceived health. In contrast, MCS ÷ *FSD* was reported to be more physically active than the control group (*p* = 0.01). Comparing median levels of biochemical markers revealed significant differences between MCS + *FSD* and the control group for insulin (*p* = 0.0005) and insulin resistance, HOMA-IR (*p* = 0.017), whereas no significant differences in median biochemical marker levels were seen when comparing the MCS ÷ *FSD* and the control group. There was a higher proportion of individuals with metabolic syndrome when comparing the MCS + *FSD* and the control group (*p* = 0.002) (Table 1).

Overall, we found no statistically significant associations for lipid metabolism markers (except triglycerides) among neither MCS + *FSD* nor MCS ÷ *FSD* compared with the controls, and excluding the statin users and the individuals with diabetes did not change the risk estimates (Table 2, Table 3, Table 4 and Table 5). 

We found that MCS + *FSD* was associated with a higher level of triglycerides, glucose, and HbA1c, though this seemed to be explained by physical activity, BMI, and waist circumference. Similarly, we found higher levels of insulin and HOMA-IR, which, to a lower degree, could be explained by the variables that were adjusted for (Table 2). Excluding statin users and individuals with diabetes showed similar associations for triglycerides, HbA1c, insulin, and HOMA-IR, though the associations were no longer significant in the adjusted model (Table 3). 

Correspondingly, we found higher levels of HbA1c, insulin, and HOMA-IR among MCS ÷ *FSD* compared to the control group. The covariates did not fully explain the differences for insulin and HOMA-IR (Table 4). Excluding statin users and individuals with diabetes strengthened the associations with glucose, HbA1c, insulin, and HOMA-IR, which could not be explained by physical activity, BMI, and waist circumference (Table 5). 

The further adjustment of Cohens perceived stress did not change the risk estimates of any associations for either the lipid or glucose metabolism markers (Table S1). In the sensitivity analyses, further adjustment for known heart diseases as well as for depression, anxiety, self-perceived health status, and self-perceived physical fitness did not change any of the risk estimates in either MCS + *FSD* or MCS ÷ *FSD* (Table S2). There was indication of the effect modification caused by chronic mental stress on the total cholesterol and HbA1c in MCS ÷ *FSD* compared to the controls (Table S3).

**Figure 1:**
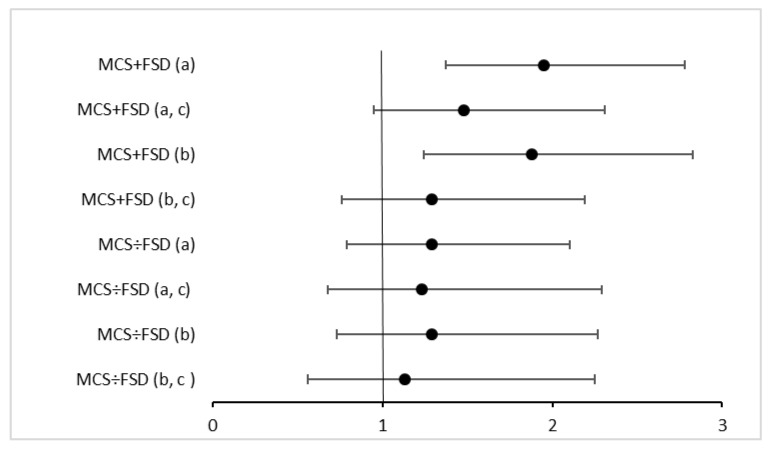
Odds ratio of metabolic syndrome among MCS + *FSD* and MCS ÷ *FSD* compared to controls. (a) Individuals with diabetes excluded and adjusted for age, sex, age^2^. (b) Individuals with diabetes and statin users excluded and adjusted for age, sex, age^2^. (c) Additionally adjusted for BMI and physical activity.

We found higher odds of metabolic syndrome in MCS + *FSD* compared to the controls, which seemed to be able to be explained by physical activity and BMI. There was no statistically significant difference in the odds of metabolic syndrome in MCS ÷ *FSD* compared to the controls in either of the adjusted models (Figure 1). 

## 4. Discussion

In this general population-based cohort study, we found no differences in the markers for the lipid metabolism or for the odds of metabolic syndrome between MCS individuals and controls with no *FSD* comorbidities. However, we found that MCS ÷ *FSD* had elevated glucose and insulin levels, had higher long-term blood glucose, and were more insulin resistant compared to the controls. This association was not affected by any additional adjustments for lifestyle covariates. Both higher and lower prevalence have been reported in other studies, and these differences are most likely due to differences in study design and disease definitions across studies, and the true prevalence is still unknown.

We did not detect any associations between MCS and altered levels of cholesterol biomarkers but did observe elevated triglyceride levels in MCS + *FSD* compared to the controls. This seemed to be explained by physical activity level, BMI, and waist circumference. We are not aware of other studies that looked at lipid markers in MCS cases. 

MCS + *FSD* were more likely to be insulin resistant compared to the controls, and adjusting for physical activity, waist circumference, and BMI did not change the observed association. However, the association disappeared in the adjusted model when the individuals with diabetes and the statin users were excluded. Insulin sensitivity is closely related to obesity, and two of the strongest independent risk factors are waist circumference and body fat percentage [35,36,37], which maybe is more pronounced in individuals with diabetes. This has been explained by the ability of adipose tissue to secrete free fatty acids and adipocytokines, which can thus interfere with the insulin-signaling system [38,39]. However, among MCS ÷ *FSD*, the association with insulin resistance compared to the controls persisted and could not be explained by physical activity, BMI, or waist circumference nor diabetic individuals or statin users. It has been shown that exposure to environmental pollutants can affect both the function and survival of pancreas beta-cells, insulin release, and glucose provision [40,41], and environmental chemicals have been associated with several of the mechanisms that are involved in obesity [42]. If MCS individuals are more vulnerable to endocrine disruptors, they could also be more susceptible to negative effects in different metabolism processes. However, a previous study including women aged 30–64 years old found no indication of higher insulin or insulin resistance among 223 MCS cases compared to 194 controls [16]. Inconsistency in results could be explained to some extent by the fact that other *FSD*s among MCS cases were not taken into consideration in the study by Baines et al. Moreover, the size of and thus the limited variation in the control group and relatively high insulin levels in both cases and controls could also be relevant differences between studies [16]. 

Elevated levels of glucose and long-term blood glucose (HbA1c) persisted in MCS ÷ *FSD* and not in MCS + *FSD* in the fully adjusted model excluding persons with diabetes and statin users; thus, the MCS ÷ *FSD* phenotype seems to differentiate between glucose and long-term blood glucose from the MCS + *FSD* phenotype. We found no other studies exploring glucose in MCS cases compared to controls except for the study conducted by Baines et al., who did not find differences in the mean glucose levels, which could be explained by the same issues mentioned above [16]. One could speculate as to whether the observed disturbances in glucose metabolism in MCS ÷ *FSD* is due to higher oxidative stress in this group compared to the controls. Oxidative stress is a phenomenon that is caused by an imbalance between the production and accumulation of oxygen reactive species in cells and tissues and the ability of a biological system to detoxify these reactive products [43]. Oxidative stress is suggested to be responsible, with different degrees of importance, for the onset and/or progression of several diseases, such as cancer, diabetes, metabolic disorders, atherosclerosis, and cardiovascular diseases [43,44]; thus, this might also be involved in the metabolic imbalances observed in MCS. Moreover, oxidative stress has previously been suggested to be important in the onset of MCS [45]. Exposure to environmental pollutants and chemical solvents (components in symptom triggers of MCS) contribute to enhanced exogenous free radical production, and when these exogenous compounds penetrate the body, they are degraded or metabolized, and free radicals are generated as by-products, resulting in the possibility of oxidative stress occurring [43]. Although the impairment of the chemical defensive system in MCS has been hypothesized [45], we do not have sufficient knowledge to answer questions such as why MCS ÷ *FSD* individuals are more vulnerable to environmental pollutants that fuel oxidative stress than controls and why this difference was not seen among MCS + *FSD*. We were not able to find references that have explored such differences in other MCS or *FSD* study populations. 

Chronic mental stress, which has previously been reported in MCS and *FSD* individuals [46,47], has an impact on essential physiological processes such as disturbances in the glucose metabolism [48]. Mental stress is, among other things, responsible for the production of endogenous free radicals [44], which may induce oxidative stress, as explained above, though the level of Cohens perceived stress was within the same range in both MCS + *FSD* and MCS ÷ *FSD*. Adjustment for chronic mental stress, including anxiety and depression, did not change the observed associations, but we did find a significant effect modification of Cohens perceived stress on total cholesterol and HbA1c in MCS ÷ *FSD* compared to the controls. The rather low effect size may question the clinical significance of these findings.

Finally, regarding insulin resistance, the observed glucose metabolism dysfunction in MCS ÷ *FSD* individuals compared to the controls may simply be the result of a different lifestyle in this population in terms of sedentarism, poor sleep quality, and alcohol habits compared to the controls. However, a previous DanFunD study found that this lifestyle was more pronounced in MCS individuals with other *FSD* comorbidities than it was among MCS ÷ *FSD* when compared to individuals without MCS [2]. In our study, though based on self-reports, MCS ÷ *FSD* reported being more physically active compared to the controls. If the results are a matter of lifestyle, it would be important that health care professionals emphasize the relevance of physical activity and a healthy balanced diet in accordance with national guidelines in MCS individuals both with and without *FSD* comorbidities.

In contrast to previous studies on other *FSD*s, we found no association between MCS + *FSD* and metabolic syndrome in the adjusted models. In a US community-based study, women aged 21–49 years old with fibromyalgia (*n* = 109) were five times more likely than age-matched healthy controls (*n* = 46) to have metabolic syndrome [17], and in a population-based case–control study conducted in Georgia, United States, chronic fatigue syndrome (*n* = 111 cases vs. 123 controls) was associated with having a metabolic syndrome [18]. A significantly higher odds ratio of metabolic syndrome in IBS was also observed in a Japanese population-based cohort study that compared 213 individuals with IBS and 883 individuals without IBS after adjusting for relevant confounding factors [19]. We did see a tendency of an association between MCS and metabolic syndrome, which could be due to *FSD* comorbidity among MCS *+ *FSD*,* whereas MCS ÷ *FSD* may have a negative impact on physical health though possibly to a lesser extent [2]. 

### Strengths and Limitations

The strengths and limitations related to the DanFunD study have been discussed in previous publications [2,20,49]. One major strength is that the study is based on a large randomly invited sample of the general population, resulting in it comprising data of nearly 10.000 men and women covering an age span of 50 years [20]. Furthermore, *FSD* case status was determined using validated and previously applied case definitions along with the vast data material that was also collected, i.e., data from a general health examination, biochemical profiling, and comprehensive questionnaires; this cohort offers a unique source of data for the study of the MCS phenotype. We are among the first to explore metabolic markers in MCS while also taking the impact of comorbid *FSD* into account.

The MCS prevalence of 1.95% is similar to the results presented by Berg et al. (2.2%) in a comparable Danish cohort, though in this study, case selection was based on the lifestyle consequences of airborne chemical exposures and did not symptom patterns into consideration. Other studies report both lower and higher prevalences of MCS, which is likely the result of variation in the study design and the delimitation of MCS (i.e., self-reported clinician diagnosed MCS, self-report by questionniares, diagnosed by physician) [50].

A concern is the relatively low participation rate of the DanFunD study of 34% and the concern of whether the cohort is representative of the general adult population [20]. In a recent study of the DanFunD cohort, the prevalence of *FSD* and common mental disorders were found to be higher among non-responders [51]. Thus, the participants may be healthier than the non-participants, at least at the group level. If the non-participants have a higher symptom load, then we would expect higher variation across groups, suggesting that the significance of the present findings may be underestimated. 

Furthermore, it is a limitation that information about the participants’ medical records were not available and that *FSD* case status was not clinically verified using the gold standard but was merely based on questionnaire data. For instance, an Italian Expert consensus was recently published that suggested an assessment of the endocrinological system as part of the clinical practice for the diagnostic evaluation of a potential MCS patient [52]. In a recent study that also used data from the DanFunD cohort, a comparison of *FSD* cases that were identified via self-reported questionnaires vs. *FSD* cases that were clinically verified confirmed that the symptom questionnaires that are often applied do serve as a suitable screening tools for *FSD* in a study population and as a tool for transdiagnostic comparison [53]. With MCS being relatively poorly captured in registers, the validated and previously applied case definition is still one of the most reliable tools.

## 5. Conclusions

In this population-based cohort study, we found disturbances in the glucose metabolism in individuals with MCS compared to the controls. We found that the MCS ÷ *FSD* group had significantly higher insulin levels and higher insulin resistance compared to the controls. Differences in other metabolic health indicators were mainly driven by the coexistence of other *FSD*s. Moreover, we found that MCS cases who did not have one or more comorbid *FSD*s had higher levels of fasting as well as long term blood glucose compared to the general population. Our results could not support differences in the evaluated lipid metabolism markers or support an increased risk of metabolic syndrome in individuals with MCS compared to controls.

## Figures and Tables

**Table 1:** Characteristics of participants (median, p5–p95 unless otherwise stated).

	Controls (*n* = 7791)	MCS + *FSD* (*n* = 188)	*p*-Value	MCS ÷ *FSD* (*n* = 109)	*p*-Value
Age	54 (26–70)	56 (25–70)	0.23	57 (24–70)	0.04
Sex (% women)	51	67	<0.0001	61	0.03
Anthropometry					
Weight (kg)	75.9 (54.7–105)	74.6 (52.7–112)	0.98	73.1 (52.0–111)	0.44
Height (cm) (mean, SD)	172 (9.4)	169 (0.08)	<0.001	170 (7.8)	0.004
BMI (kg/m^2^) (mean, SD)	25.9 (4.4)	27.3 (5.78)	0.001	26.4 (5.0)	0.35
Waist circumference (cm)	88.0 (69.5–112)	90.0 (67.8–120)	0.06	88.6 (67.8–115)	0.40
Fat percentage	28.6 (15.4–44.4)	33.9 (18.0–48.3)	<0.0001	31.6 (17.2–46.3)	0.003
BMI groups (%)			<0.0001		0.04
Normal weight	47.0	41.0		46.8	
Overweight	37.2	30.8		29.4	
Obese (class I-III)	15.7	28.2		23.8	
Physical activity METS (mean, SD)	39.9 (4.28)	40.2 (4.66)	0.39	40.9 (4.88)	0.01
Cohens Stress scale	9 (1–19)	13 (3–25)	<0.0001	12 (2–22)	0.0001
SCL Anxiety	1 (0–7)	4 (0–16)	<0.0001	2 (0–13)	<0.0001
SCL Depression	2 (0–12)	6 (0–27)	<0.0001	4 (0–19)	<0.0001
High self-perceived health (%)	56	28	<0.0001	38	0.0003
High self-perceived fitness (%)	44	26	<0.0001	38	0.21
Biochemical markers					
Total cholesterol (mmol/L) (mean, SD)	5.32 (1.05)	5.32 (1.14)	0.99	5.48 (1.18)	0.13
HDL-cholesterol (mmol/L)	1.41 (0.89–2.25)	1.42 (0.85–2.24)	0.94	1.47 (0.85–2.21)	0.26
Non-HDL-cholesterol (mmol/L) (mean, SD)	3.86 (1.05)	3.84 (1.16)	0.86	3.97 (1.25)	0.33
Triglycerides (mmol/L)	1.03 (0.54–2.59)	1.09 (0.56–2.66)	0.09	1.08 (0.54–2.54)	0.30
Glucose (mmol/L)	5.40 (4.6–6.8)	5.4 (4.6–7.2)	0.29	5.4 (4.6–7.2)	0.66
HbA1c (mmol/mol) (mean, SD)	35.9 (5.39)	36.5 (6.05)	0.23	36.6 (7.04)	0.33
Insulin (pmol/L)	48.9 (19.0–150)	57.8 (21.3–202)	0.005	52.9 (23.2–176)	0.16
HOMA-IR ^a^	1.70 (0.61–6.04)	1.90 (0.65–7.77)	0.017	1.84 (0.69–6.99)	0.21
Individuals with diabetes ^b^ (%)	4	3	0.29	3	0.63
Individuals with metabolic syndrome ^c^ (%)	19	28	0.002	23	0.27

MCS, multiple chemical sensitivity, with (+) and without (÷) *FSD*, functional somatic disorders. Tested median/mean differences between MCS + *FSD* and MCS ÷ *FSD* and controls; continuous variables were tested using Kruskal–Wallis or T-test; categorical variables were tested using the Chi-squared test. ^a^ The homeostasis model of insulin resistance [29]. ^b^ Self-reported by the question “Did a doctor ever tell you that you have diabetes”. ^c^ Metabolic syndrome defined by The American Heart Association [34].

**Table 2:** Associations between markers of lipid and glucose metabolism and MCS + *FSD* compared to controls. Beta-estimates (95% CIs) were obtained by multiple linear regression with each marker as an dependent variable and with age, sex, physical activity, BMI, and waist circumference as co-variates.

	*n* Cases/Total *n*	% Difference (95% CI)	
		Model 1	Model 2
Total cholesterol	186/7950	−1 (−4, 2)	−1 (−4, 1)
HDL-cholesterol	186/7952	−2 (−6, 1)	1 (−3, 4)
Non HDL-cholesterol	183/7890	0 (−4, 4)	−2 (−6, 1)
Triglycerides	186/7952	12 (2, 22) *	3 (−5, 12)
Glucose	179/7590	2 (0, 4) *	1 (−1, 3)
HbA1c	178/7419	2 (1, 4) *	1 (0, 3)
Insulin	172/7314	42 (28, 57) *	23 (14, 34) *
HOMA-IR	171/7295	53 (37, 71) *	33 (21, 46) *

Model 1 adjusted for age and sex. Model 2 additionally adjusted for physical activity, BMI, and waist circumference. * *p*-value < 0.05. Individuals with diabetes excluded from analyses on glucose, HbA1c, insulin, HOMA-IR.

**Table 3:** Associations with markers of lipid and glucose metabolism in MCS + *FSD* compared to controls excluding statin users and individuals with diabetes. Beta-estimates (95% Cis) were obtained by multiple linear regression, with each marker as a dependent variable and with age, sex, physical activity, BMI, and waist circumference as co-variates.

	*n* Cases/Total *n*	% Difference (95% CI)	
		Model 1	Model 2
Total cholesterol	157/6581	0 (−3, 3)	−1 (−4, 2)
HDL-cholesterol	157/6582	−3 (−7, 1)	−1 (−4, 3)
Non HDL-cholesterol	155/6543	1 (−2, 5)	−1 (−5, 3)
Triglycerides	157/6582	12 (2, 23) *	6 (−3, 16)
Glucose	155/6562	1 (−1, 3)	1 (−1, 2)
HbA1c	155/6414	2 (0, 4) *	1 (0, 3)
Insulin	149/6322	24 (10, 39) *	5 (−5, 15)
HOMA-IR	148/6308	29 (13, 46) *	9 (−2, 21)

Model 1 adjusted for age and sex. Model 2 additionally adjusted for physical activity, BMI, waist circumference. * *p*-value < 0.05.

**Table 4:** Associations between markers of lipid and glucose metabolism and MCS ÷ *FSD* compared to controls. Beta-estimates (95% CIs) were obtained by multiple linear regression, with each marker as dependent variable and with age, sex, physical activity, BMI, and waist circumference as co-variates.

	*n* Cases/Total *n*	% Difference (95% CI)	
		Model 1	Model 2
Total cholesterol	108/7872	2 (−2, 5)	1 (−3, 4)
HDL-cholesterol	108/7874	0 (−5, 5)	1 (−4, 5)
Non HDL-cholesterol	106/7813	3 (−2, 8)	1 (−4, 6)
Triglycerides	108/7874	7 (−5, 20)	5 (−6,16)
Glucose	105/7516	2 (0, 4)	2 (0, 4)
HbA1C	103/7529	2 (0, 4) *	2 (0, 4)
Insulin	99/7241	20 (4, 38) *	13 (0, 28) *
HOMA-IR	99/7223	27 (8, 48) *	25 (10, 42) *

Model 1 adjusted for age and sex. Model 2 additionally adjusted BMI, physical activity, waist circumference. * *p*-value < 0.05. Individuals with diabetes excluded from analyses for glucose, HbA1c, insulin, HOMA-IR.

**Table 5:** Associations between markers of lipid and glucose metabolism and MCS ÷ *FSD* compared to controls, excluding statin users and individuals with diabetes. Beta-estimates (95% CIs) were obtained by multiple linear regression, with each marker as a dependent variable and with age, sex, physical activity, BMI, and waist circumference as co-variates.

	*n* Cases/Total *n*	% Difference (95% CI)	
		Model 1	Model 2
Total cholesterol	96/6677	2 (−1, 6)	1 (−2, 5)
HDL-cholesterol	96/6378	−1 (−6, 4)	−1 (−5, 4)
Non HDL-cholesterol	94/6637	4 (−1, 9)	2 (−2, 7)
Triglycerides	96/6678	9 (−4, 24)	8 (−3, 21)
Glucose	96/6658	2 (0, 5) *	2 (0, 5) *
HbA1c	95/6666	3 (1, 5) *	3 (1, 5) *
Insulin	91/6413	24 (7, 42) *	15 (2, 30) *
HOMA-IR	91/6399	32 (13, 54) *	29 (14, 46) *

Model 1 adjusted for age and sex. Model 2 additionally adjusted for BMI, physical activity, waist circumference. * *p*-value < 0.05.

## Data Availability

Data cannot be made publicly available for ethical and legal reasons. Public availability may compromise participant privacy, and this would not comply with Danish legislation. Access to the subset of data included in this study can be gained through submitting a request to The Capital Region Knowledge Center for Data Compliance, The Capital Region Denmark; cru-fp-vfd@regionh.dk. Acquisition of data is only allowed after permission to handle data has been obtained in accordance with the guidelines stated by the Danish Data Protection Agency: http://www.datatilsynet.dk/english (accessed on 21 November 2014).

## Supplementary Materials

The following are available online at https://www.mdpi.com/article/10.3390/ijerph182312654/s1, Table S1: Associations with markers of lipid and glucose metabolism in MCS + *FSD* and MCS ÷ *FSD*, compared to controls; adjusted for Cohens Perceived Stress scale. Table S2: Sensitivity analysis including additional adjustment for known heart diseases, depression, anxiety, self-perceived health status, and self-perceived physical fitness on the associations between lipid and glucose metabolism markers in MCS + *FSD* and MCS ÷ *FSD* compared to the controls. Table S3: Effect modification of Cohens Perceived Stress scale on the associations with lipid and glucose metabolism markers in MCS + *FSD* and MCS ÷ *FSD* compared to the controls.
