# Supplementary material for: Insulin Resistance Is Associated with Multiple Chemical Sensitivity in a Danish Population-Based Study—DanFunD
Source: Int J Environ Res Public Health. 2021 Nov 30;18(23):12654. doi: 10.3390/ijerph182312654 (PMC8657139; doi:10.3390/ijerph182312654)
Supplement: Supplementary file 1 [file ijerph-18-12654-s001.zip › ijerph-1462800-supplementary.pdf]

Supplementary Table S1

Associations with markers of lipid and glucose metabolism in MCS + *FSD* and MCS ÷*FSD*, compared to the controls; adjusted for Cohens Perceived Stress scale.

	**MCS +*FSD***		**MCS ÷*FSD***	
	**n cases/total n**	**% difference (95% CI) [a]**	**n cases/total n**	**% difference (95% CI) [a]**
**Total cholesterol**	186/7950	-1 (-4, 2)	157/6581	1 (-2, 5)
**HDL-cholesterol**	186/7952	1 (-2, 5)	157/6582	0.9 (-4, 6)
**Non HDL-cholesterol**	183/7890	-2 (-6, 1)	155/6543	1 (-4, 6)
**Triglycerides**	186/7952	2 (-6, 11)	157/6582	4 (-7, 16)
**Glucose**	179/7590	1 (-1, 3)	155/6562	2 (-1, 4)
**HbA1c**	178/7419	1 (-1, 3)	155/6414	2 (0.5, 4)*
**Insulin**	172/7314	24 (14, 35)*	149/6322	14 (0.7, 28)*
**HOMA-IR**	171/7295	34 (22, 47)*	148/6308	25 (10, 42)*

a) Adjusted for age, sex, physical activity, BMI, waist circumference, and Cohens perceived stress scale.
*p-value <0.05. Diabetes cases are excluded from analyses on glucose, HbA1c, insulin, HOMA-IR.

Supplementary Table S2

Sensitivity analysis including additional adjustment for known heart diseases, depression, anxiety, self-perceived health status, and self-perceived physical fitness on the associations between lipid and glucose metabolism markers in MCS +*FSD* and MCS ÷*FSD* compared to the controls.

	**MCS ÷*FSD***		**MCS ÷*FSD***	
	**n cases/total n**	**% difference (95% CI) [a]**	**n cases/total n**	**% difference (95% CI) [a]**
Total cholesterol	157/6581	-1 (-4, 2)	96/6677	1 (-2, 5)
HDL-cholesterol	157/6582	0.1 (-4, 4)	96/6378	-1 (-5, 4)
Non HDL-cholesterol	155/6543	-2 (-5, 2)	94/6637	2 (-2, 7)
Triglycerides	157/6582	4 (-4, 14)	96/6678	7 (-5, 20)
Glucose	155/6562	0.2 (-2, 2)	96/6658	3 (0.3, 5)*
HbA1c	155/6414	1 (-1, 3)	97/6668	3 (1, 5)*
Insulin	149/6322	4 (-6, 15)	91/6413	14 (1, 28)*
HOMA-IR	148/6308	6 (-5, 18)	91/6399	25 (10, 42)*

a) Adjusted for age, sex, BMI, physical activity, waist circumference, known heart diseases as well as depression, anxiety, self-perceived health status, and self-perceived physical fitness. *$p<0.05$. Diabetes individuals and statin users were excluded from all analyses.

Supplementary Table S3

Effect modification of Cohens Perceived Stress scale on the associations with lipid and glucose metabolism markers in MCS +*FSD* and MCS ÷*FSD*
compared to the controls.

	**MCS +*FSD***			**MCS ÷*FSD***		
	**n cases/total n**	**% difference (95% CI) [a]**	**Test for interaction of Cohens perceived stress scale, p-value**	**n cases/total n**	**% difference (95% CI) [a]**	**Test for interaction of Cohens perceived stress scale, p-value**
Total cholesterol	157/6581	0.2 (-1, 0.6)	0.27	96/6677	0.7 (0.1, 1)	0.01
HDL-cholesterol	157/6582	-1 (-2, 1)	0.05	96/6378	-1 (-1, 0.7)	0.81
Non HDL-cholesterol	155/6543	0.5 (0.0, 1)	0.07	94/6637	1 (0.2, 2)	0.01
Triglycerides	157/6582	-1 (-2, 0.8)	0.43	96/6678	-1 (-3, 2)	0.93
Glucose	155/6562	0.1 (-1, 0.4)	0.54	96/6658	0.2 (-1, 0.6)	0.27
HbA1c	155/6414	0.2 (-1, 0.4)	0.14	95/6666	0.4 (0.0, 0.7)	0.03
Insulin	149/6322	0.0 (-2, 2)	0.95	91/6413	-1 (-4, 2)	0.51
HOMA-IR	148/6308	0.3 (-2, 2)	0.77	91/6399	0.2 (-3, 3)	0.89

a) Effect of Cohens perceived stress scale on biomarker levels in MCS individuals vs controls adjusted for sex and age.
Diabetes individuals and statin users were excluded from all analyses.
